# Large variability in response to future climate and land‐use changes among Chinese Theaceae species

**DOI:** 10.1002/ece3.9480

**Published:** 2022-11-15

**Authors:** Junfeng Tang, Xuzhe Zhao

**Affiliations:** ^1^ Key Laboratory of Southwest China Wildlife Resources Conservation (Ministry of Education), Institute of Ecology China West Normal University Nanchong China

**Keywords:** conservation strategies, ensemble species distribution models, isolated impact, modulating effect, Theaceae species

## Abstract

Theaceae is an important family in the phylogeny of angiosperm in China, which are potentially threatened by future changes in climatic and land use conditions. Therefore, understanding and predicting the isolated and combined effects of these two global change factors on Theaceae species is crucial for biodiversity conservation. Here, we assessed the isolated and combined effects of climate and land use change on the distribution shifts of 95 Theaceae species under different future scenarios by comparing projections of three model configurations: (1) dynamics climate and constant land use variables; (2) constant climate and dynamics land use variables; and (3) dynamics climate and dynamics land use variables. We find that all the three types of models predicted range contractions for most of the 95 Theaceae species under all future scenarios. Moreover, we find that climate change has rather strong effect for most species while land use change has nonsignificant or weak effect on future species distributions, although both of these two isolated effects are highly variable across individual species. Finally, the combined effect of these two factors reveals that the land use change may amplify or buffer distribution shifts expected from climate change impact depending on species. These findings emphasize the importance of taking into account the large variability in response to land use change among Theaceae species when developing land‐based conservation strategies in a changing climate.

## Graphical abstract

The modulating effect of land use change within climate scenarios is complex, that is, land use change may amplify or buffer distribution shifts expected from climate change impact depending on species. These findings highlight the importance of taking into account the large variability in response to land use change among Theaceae species when developing land‐based conservation strategies in a changing climate.

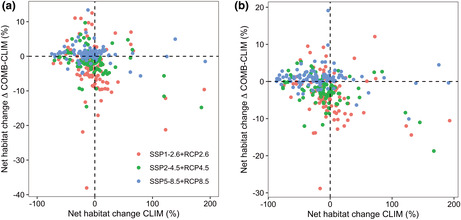

## 1 INTRODUCTION

Climate change and land use change are currently two of the most important factors driving the distributional shifts for many species (Newbold, 2018; Powers & Jetz, 2019; Sala et al., 2000). Over the past decades, the isolated effects of these two global change factors on species' range shifts have been extensively assessed, which is expected to be negative or positive for individual species (e.g., Chen et al., 2011; Powers & Jetz, 2019; Tang & Zhao, 2022). However, it has become increasingly clear that these two factors do interact to drive shifts in the location of suitable habitat of a species, rather than act separately from one another (Auffret & Thomas, 2019). Moreover, recent studies suggest that the interacting effects of climate and land‐use change are complex and species‐specific, for they could either amplify or buffer the isolated impact of a single factor for a certain species (Auffret & Thomas, 2019; Radinger et al., 2016). Therefore, to avoid further biodiversity loss, conservationists need to identify species likely to be vulnerable to global change, which requires improved estimations of species' range shifts under the combination of climate and land use change scenarios (Jarvie et al., 2022).

The family Theaceae forms a major component of tropical and subtropical ecosystems in Asia (Wu, 1995; Zhang et al., 2020). In China, it is also an important angiosperm family comprising about 12 genera and 274 species (Min & Zhang, 1996; Shen et al., 2016). These Theaceae species are mainly distributed in southern China, including Yunnan, Guizhou, Guangxi, Guangdong, Sichuan, Chongqing, Hubei, Hunan, Fujian, Zhejiang, and Taiwan Provinces (Grote & Dilcher, 1989; IUCN, 2017). The populations of these Theaceae species are often found in tropical and subtropical moist lowland or mountaine with ample amounts of water and prefer acidic soils (Ming, 2000). Moreover, Theaceae is also an economically important family in China, for many Theaceae species can be used for eating, drinking, ornamental, wood utilization, skin care, beauty, and fertilizer (Shen et al., 2010). Despite their importance, over 33% are currently at risk of extinction on the Red List of Threatened Species™, primarily due to changes triggered by urbanization and other forms of land‐use change (IUCN, 2017). More importantly, due to the ongoing and continuing land use change, these threatened species together with their ecosystem services will face severe challenges (Rao et al., 2018).

Besides, climate change is expected to exacerbate these current threats to Theaceae species. Species distribution models (SDMs) have predicted that most of Theaceae species will experience dramatic loss of their suitable habitat and some additional species will become threatened by 2070s (Zhang et al., 2020). Moreover, there is large variability among individual species' responses to future climate change (Zhang et al., 2020). However, an improved understanding of how the distribution of an individual species shifts under global change requires more detailed analyses by incorporating land use variables into climate SDMs (Prestele et al., 2021; Ramachandran et al., 2020), which has important implications for the protection of Theaceae species. For example, understanding the combined effect of climate change and land use change on the future distribution of a target species may help to design more targeted land‐management policies to offset the negative effects of climate change (Liu et al., 2022; Marshall et al., 2018; Prestele et al., 2021; Ramachandran et al., 2020; Tang et al., 2020; Tang & Zhao, 2022). Despite that, no studies have assessed the interacting effects of climate change and land use change on the future distributional changes of Theaceae species, especially the modulating effect of land use change within climate scenarios. Furthermore, whether there is large variability in response to these two global change factors across Chinese Theaceae species is still poorly understood.

To fill these knowledge gaps, in this study, we aim to (a) quantify distributional shifts on individual species due to isolated climate and land‐use change effects and (b) assess the modulating effect of land use change on changes in suitable habitat area within different climate change scenarios. To do so, we compared projected distributions by three types of models: (i) dynamics climate and constant land use models (CLIM); (ii) constant climate and dynamics land use models (LU); and (iii) dynamics climate and dynamics land use models (COMB). We predicted that these three types of models would yield different projections for the distribution patterns of Theaceae species and the modulating effect of land use change within climate change scenarios was significant.

## 2 MATERIALS AND METHODS

### 2.1 Study area and species occurrence data

**FIGURE 1:**
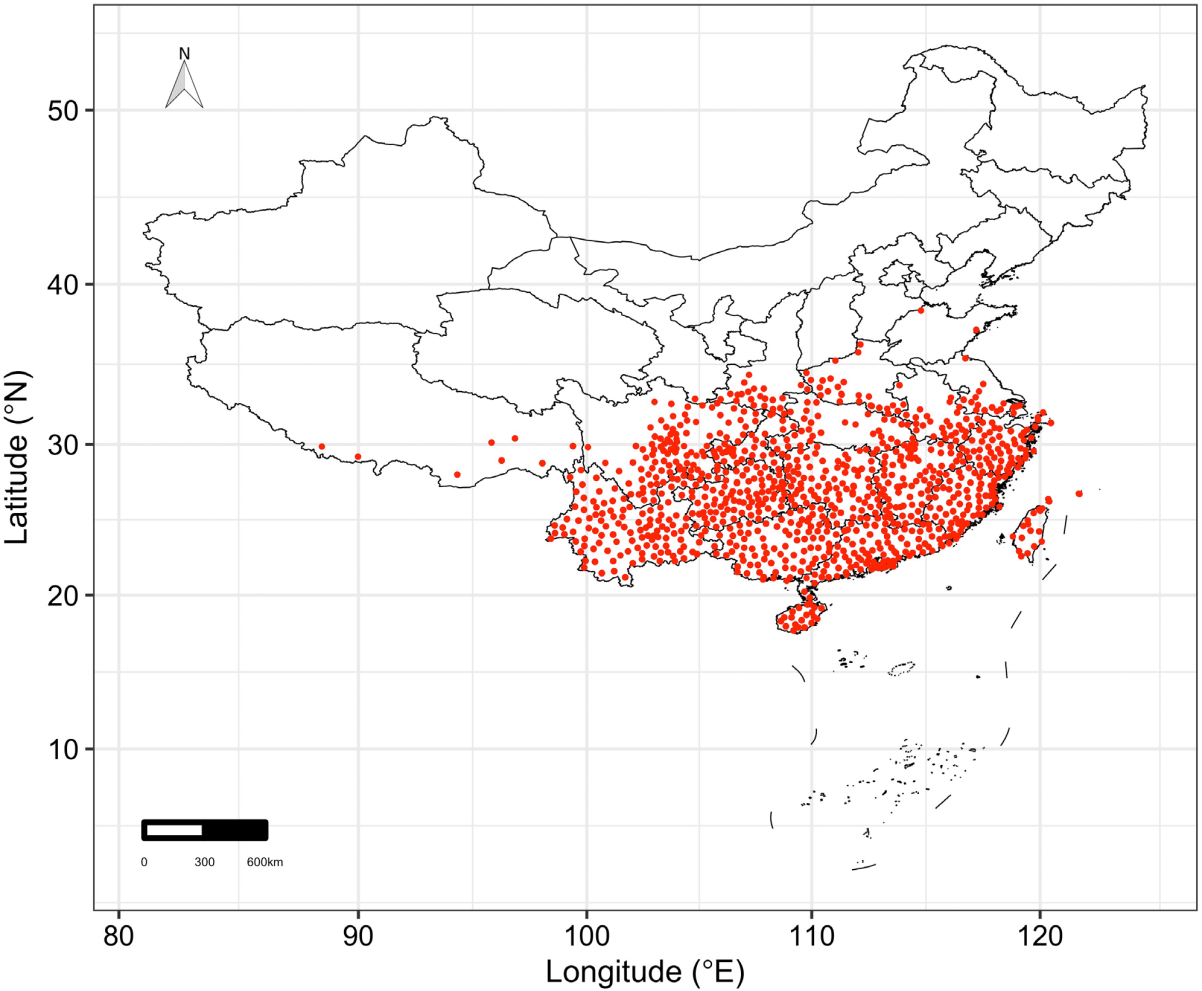
The occurrence records (red points) of the 95 Theaceae species used in this study.

This study was conducted in the entirety of China, which harbors ~46% (274 species) of total Theaceae species worldwide (Zhang et al., 2020). The occurrence records for the Theaceae species in the study area were obtained from the data set compiled and cleaned by Zhang et al. (2020). This data set contains 12,055 occurrence records for 200 Theaceae species, with the original species occurrence data collected from Chinese Virtual Herbarium (http://www.cvh.ac.cn/) and the National Specimen Information Infrastructure (http://www.nsii.org.cn/). For modeling species distribution, we created 10 km × 10 km grid cells across the study area and all the occurrence records were overlaid onto these grid cells. For each species, we removed duplicate records within each gird cell and then removed species with <30 occurrence records. Finally, 95 Theaceae species were retained in the subsequent analysis (Figure 1).

### 2.2 Current and future climate and land use variables

To model the current distribution of the 95 Theaceae species, we downloaded the current (1970–2000) 19 bioclimatic variables from the WorldClim global climate data set with a spatial resolution of 30 arc seconds (~1 km; Fick & Hijmans, 2017), and downloaded 10 land use variables with a spatial resolution of 1 km from the FROM‐GLC database (Li et al., 2016). To project future response of these 95 Theaceae species to climate and land use change, we downloaded the same 19 bioclimatic variables under Shared Socio‐economic Pathways (SSPs) scenarios (i.e., SSP1‐2.6, SSP2‐4.5, and SSP5‐8.5) for two time periods (i.e., 2050s [2041–2060] and 2070s [2061–2080]) from the WorldClim Version 2.1 data set, with a spatial resolution of 2.5 arc minutes (~10 km). In particular, these bioclimatic variables were extracted from the most recent global circulation model developed in the sixth Assessment Report of the Intergovernmental Panel on Climate Change (IPCC6), CNRM‐CM6‐1, for this model has been recommended for use in China (Rogelj et al., 2018). Correspondingly, we also downloaded the same 10 land use variables with a spatial resolution of 1 km under three representative concentration pathways (RCP2.6, RCP4.5 and RCP8.5) for 2050s and 2070s from the FROM‐GLC database, respectively. To make all environmental variables have the same spatial resolution, we resampled all environmental layers to 10 km × 10 km.

Following Prestele et al. (2021), we used three types of variable sets to assess the isolated and combined impacts of climate and land use change on the future distribution of the 95 Theaceae species: (i) dynamics climate and constant land use models (CLIM) under three climate change scenarios (i.e., SSP1‐2.6, SSP2‐4.5, and SSP5‐8.5); (ii) constant climate and dynamics land use models (LU) under three land use change scenarios (i.e., RCP2.6, RCP4.5, and RCP8.5); and (iii) dynamics climate and dynamics land use models (COMB) under the combination of RCPs and corresponding SSPs scenarios (i.e., SSP1‐2.6 + RCP2.6, SSP2‐4.5 + RCP4.5, and SSP5‐8.5 + RCP8.5).

To avoid multicollinearity of the predictor variables, Pearson's correlation coefficients were used for variable selection. When two variables were highly correlated (|*r*| > .7; Dormann et al., 2013), we selected the variable with greater biologically meaningful for the Theaceae species into subsequent modeling. Finally, five bioclimatic variables and five land use variables were retained for future analysis. The climate variables included annual mean temperature (BIO1), Isothermality (BIO3), annual temperature range (BIO7), annual precipitation (BIO12), and precipitation seasonality (BIO15). The land use variables included the proportion of area covered by cropland (CL), forest (FL), grassland (GL), shrubland (SL), and waters (WL) in each grid cell.

### 2.3 Species distribution modeling

We adopted ensemble approaches to make current and future projections of potential suitable habitat for the 95 Theaceae species using the biomod2 package in the R software environment (version 4.1.3; R Development Core Team, 2022). The relationships between the species distribution data and the ten selected predictor variables were estimated via three modeling algorithms: generalized boosting model (Ridgeway, 1999); generalized linear model (McCullagh & Nelder, 1989) and maximum entropy model (Phillips et al., 2006). The model performance of the three algorithms were evaluated using the two most commonly used metrics: the area under the receiver operating characteristic curve (AUC) and the true skill statistic (TSS) through a fivefold cross‐validation technique. Algorithms with AUC < 0.8 and TSS < 0.5 were considered to have poor predictive ability (Allouche et al., 2006; Swets, 1988) and were thus not included in the final ensemble model. Relative importance of the ten explanatory variables from the ensemble model was determined by a randomization procedure (Thuiller et al., 2014). The final ensemble model was then projected to current climate and land use conditions. Subsequently, one habitat suitability was produced for each of the three model types and for each the climate and/or land use change scenarios at 2050s and 2070s, respectively. Finally, all these maps were converted into binary presence‐absence maps by selecting a probability threshold maximizing the TSS value (Thuiller et al., 2014).

### 2.4 Assessment of climate and land use impacts

To assess the isolated and combined impacts of climate and land use change on the future distribution of the 95 Theaceae species, we calculated the distributional changes of each species for each of the three model types under six different future scenarios (three SSP/RCP scenarios × two time periods), using the following equation:

$$\mathrm{CSH}=\frac{a-b}{b}\times 100\%,$$

where CSH is the relative change in total area of suitable habitat, and *a* and *b* are the predicted suitable habitat area under current and future periods, respectively.

## 3 RESULTS

### 3.1 Model performance and variable importance

**FIGURE 2:**
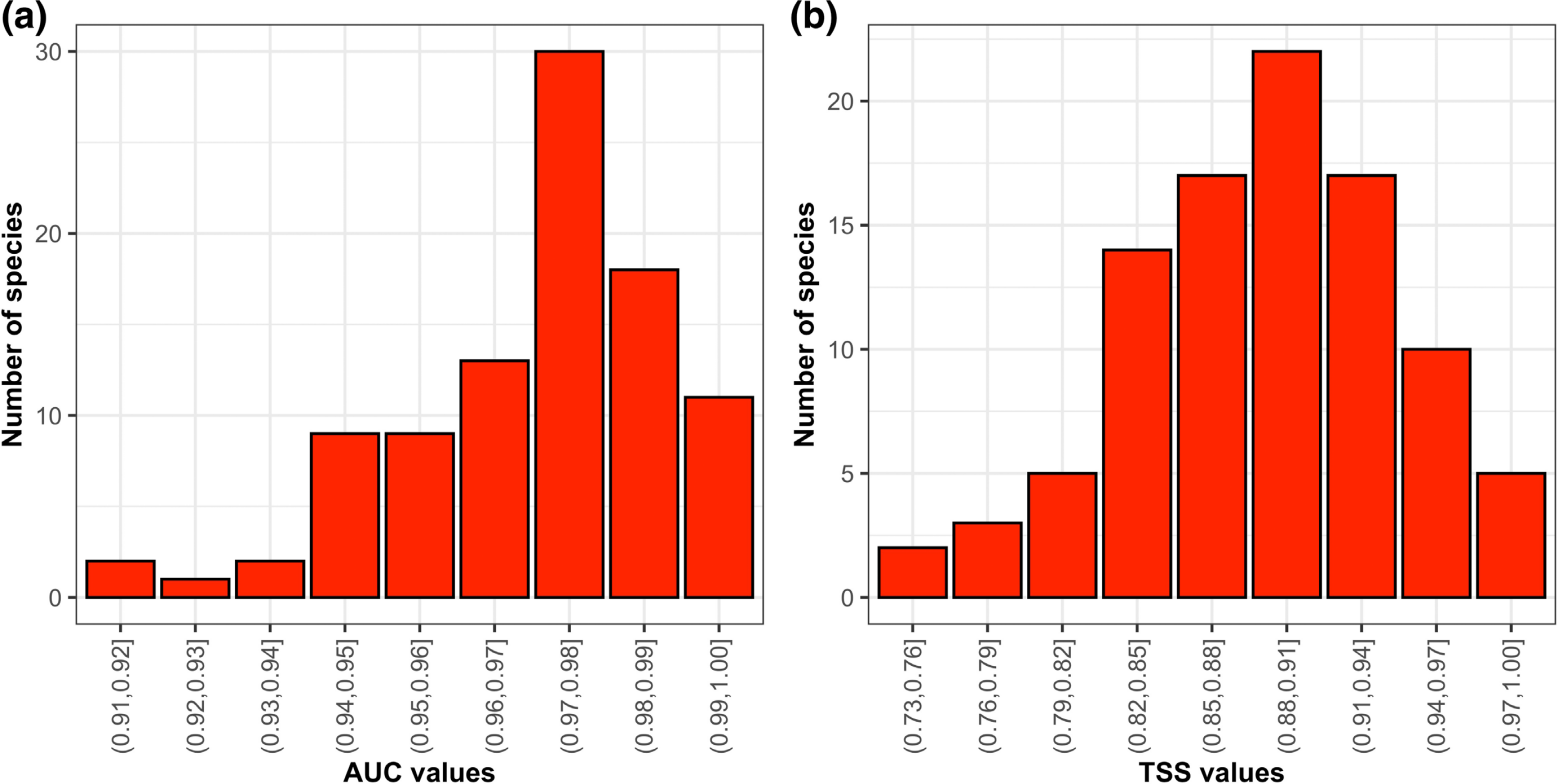
The area under the receiver operating characteristic curve (AUC; a) and the TSS (b) values of the ensemble species distribution models for the 95 Theaceae species.

The AUC and TSS values of our ensemble models for all of the 95 Theaceae species ranged from 0.911 to 0.996 and from 0.747 to 0.984, respectively, indicating that the predictive ability of our ensemble models was excellent (Figure 2). Of the ten environmental variables included in our ensemble models, temperature annual range (BIO7), mean annual temperature (BIO1), and total annual precipitation (BIO12) were the most important factors in determining the current distributions of the 95 Theaceae species, with a mean importance of 0.431 ± 0.244, 0.309 ± 0.168, and 0.300 ± 0.198, respectively, while other variables contributed little to the models (Table 1).

**TABLE 1:** The relative importance (mean ± SD) of the ten selected environmental variables in determining the current distributions of the 95 Theaceae species.

Variable	Description	Relative importance
BIO1	Annual mean temperature	0.309 ± 0.168
BIO3	Isothermality	0.079 ± 0.119
BIO7	Temperature annual range	0.431 ± 0.224
BIO12	Total annual precipitation	0.300 ± 0.198
BIO15	Precipitation seasonality	0.101 ± 0.093
CL	The proportion of area covered by cropland	0.095 ± 0.062
FL	The proportion of area covered by forest	0.054 ± 0.046
GL	The proportion of area covered by grassland	0.106 ± 0.078
SL	the proportion of area covered by shrubland	0.039 ± 0.041
WL	the proportion of area covered by waters	0.017 ± 0.023

### 3.2 The isolated effect of climate and land use on habitat suitability

**FIGURE 3:**
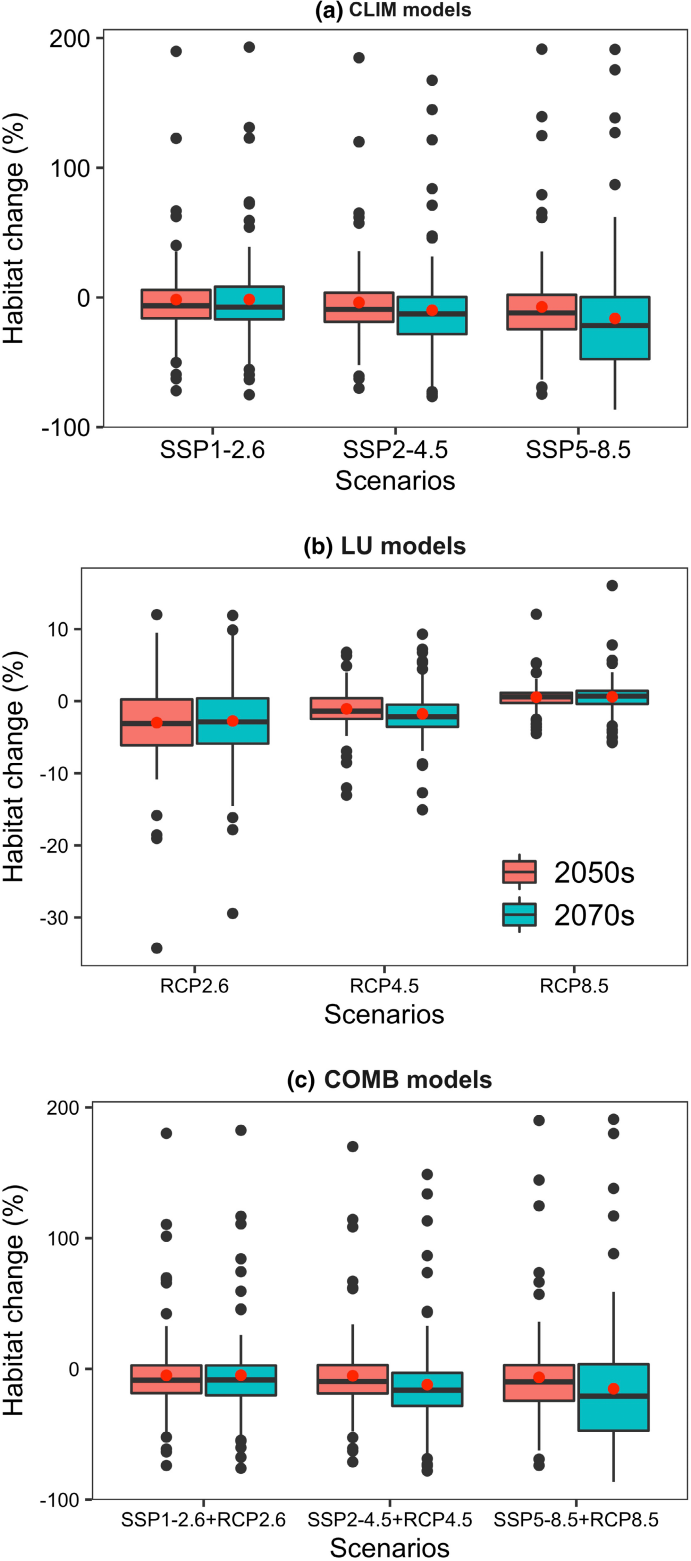
Net changes in suitable habitat for the 95 Chinese Theaceae species projected by (a) CLIM models (dynamics climate and constant land use models), (b) LU models (constant climate and dynamics land use models) and (c) COMB models (dynamics climate and land use models). The red points in (a)–(c) are the mean values of each group.

On average, the CLIM models predicted an increase of net habitat losses of the 95 Theaceae species from SSP1‐2.6 to SSP5‐8.5 for both time periods (Figure 3a). Specifically, the average relative changes in suitable habitat across the 95 Theaceae species projected by CLIM models amounts to −1.261%, −3.886%, and −7.261% for SSP1‐2.6, SSP2‐4.5, and SSP5‐8.5 by 2050s and amounts to −1.490%, −9.790% and −16.154% for SSP1‐2.6, SSP2‐4.5 and SSP5‐8.5 by 2070s (Figure 3a). Despite that, the CLIM models projected a large variation in the individual species' response to future climate change, as the relative changes in suitable habitat for many species were shown as ‘outliers’ in the boxplots (Figure 3a). Particularly, while 21 out of the 95 Theaceae species were predicted to lose >50% of their suitable habitat under the worst climate scenarios (i.e., SSP5‐8.5 by 2070s), 15 species would gain their habitat more than 10% (Figure 3a).

**FIGURE 4:**
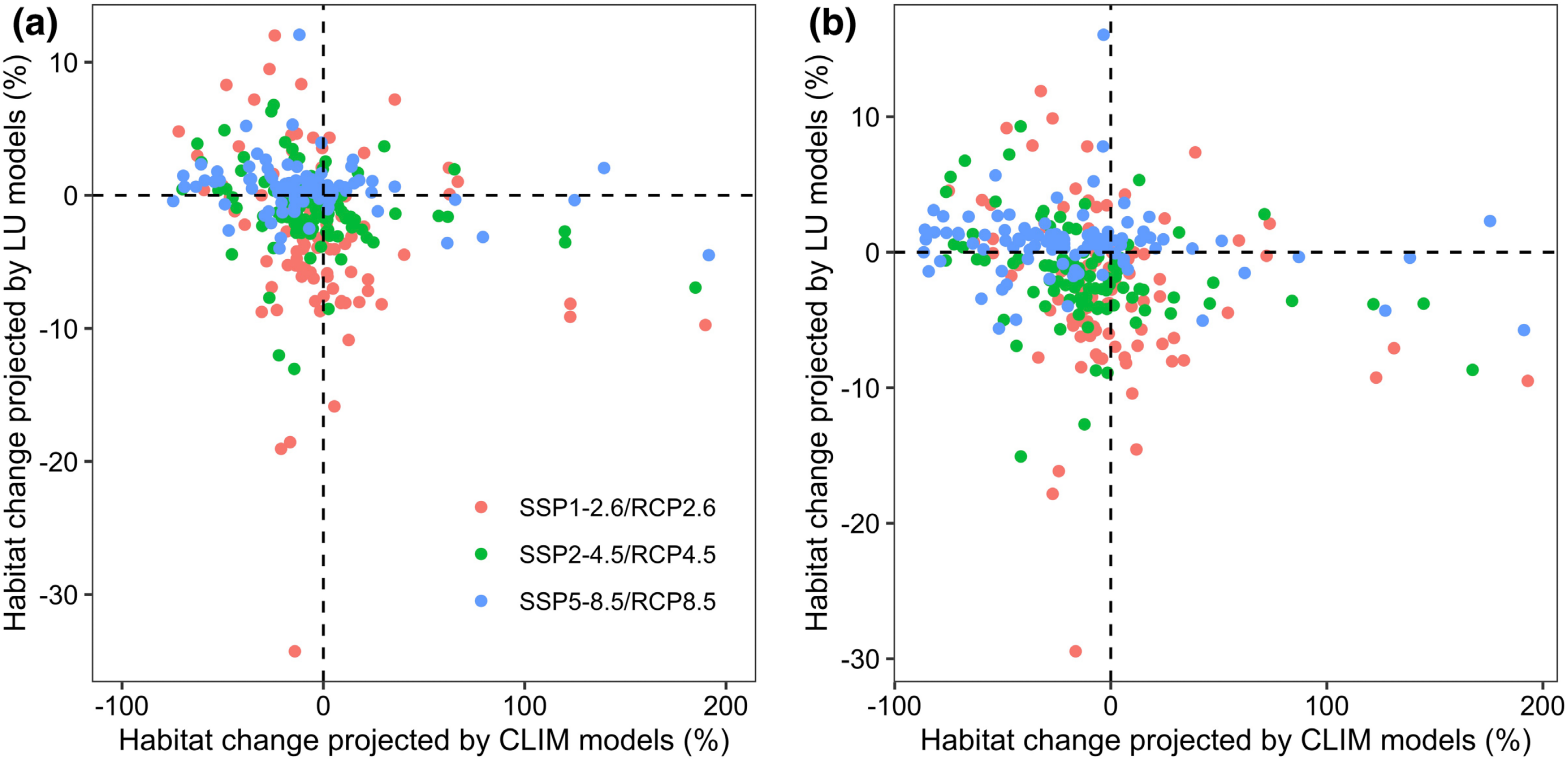
Relationship between the relative changes in suitable habitat projected by CLIM models (dynamics climate and constant land use models) and LU models (constant climate and dynamics land use models) for the 95 Theaceae species under different RCP scenarios by (a) 2050s and (b) 2070s. The points in (a)–(b) represent individual species.

On the contrary, the LU models predicted a decrease of habitat losses of the 95 Theaceae species from RCP2.6 to RCP8.5 for both time periods (Figure 3b). Specifically, the average relative changes in suitable habitat across the 95 Theaceae species projected by LU models amounts to −2.980%, −1.061%, and 0.564% for RCPs 2.6, 4.5, and 8.5 by 2050s, respectively, and amounts to −2.740%, −1.756% and 0.605% for RCPs 2.6, 4.5, and 8.5 by 2070s, respectively (Figure 3b). Besides, the magnitude of changes in suitable habitats projected by LU models for most species (at least 77 species) is smaller than that projected by CLIM models (Tables S1–S2; Figure 3). Despite that, the isolated impact of CLIM and LU on habitat suitability show large variable across the 95 Theaceae species (Figure 4). Specifically, the mean number of species that shows (i) habitat gains under all climate and land use change scenarios, (ii) habitat gains under all climate change scenarios but habitat losses under all land use change scenarios, (iii) habitat losses under all climate change scenarios but habitat gains under all land use change scenarios, and (iv) habitat losses under all climate and land use change scenarios by 2050s was 7, 25, 23, and 40, respectively, and was 4, 23, 18, and 50 by 2070s, respectively (Figure 4).

### 3.3 The modulating effect of land use change within climate scenarios

**FIGURE 5:**
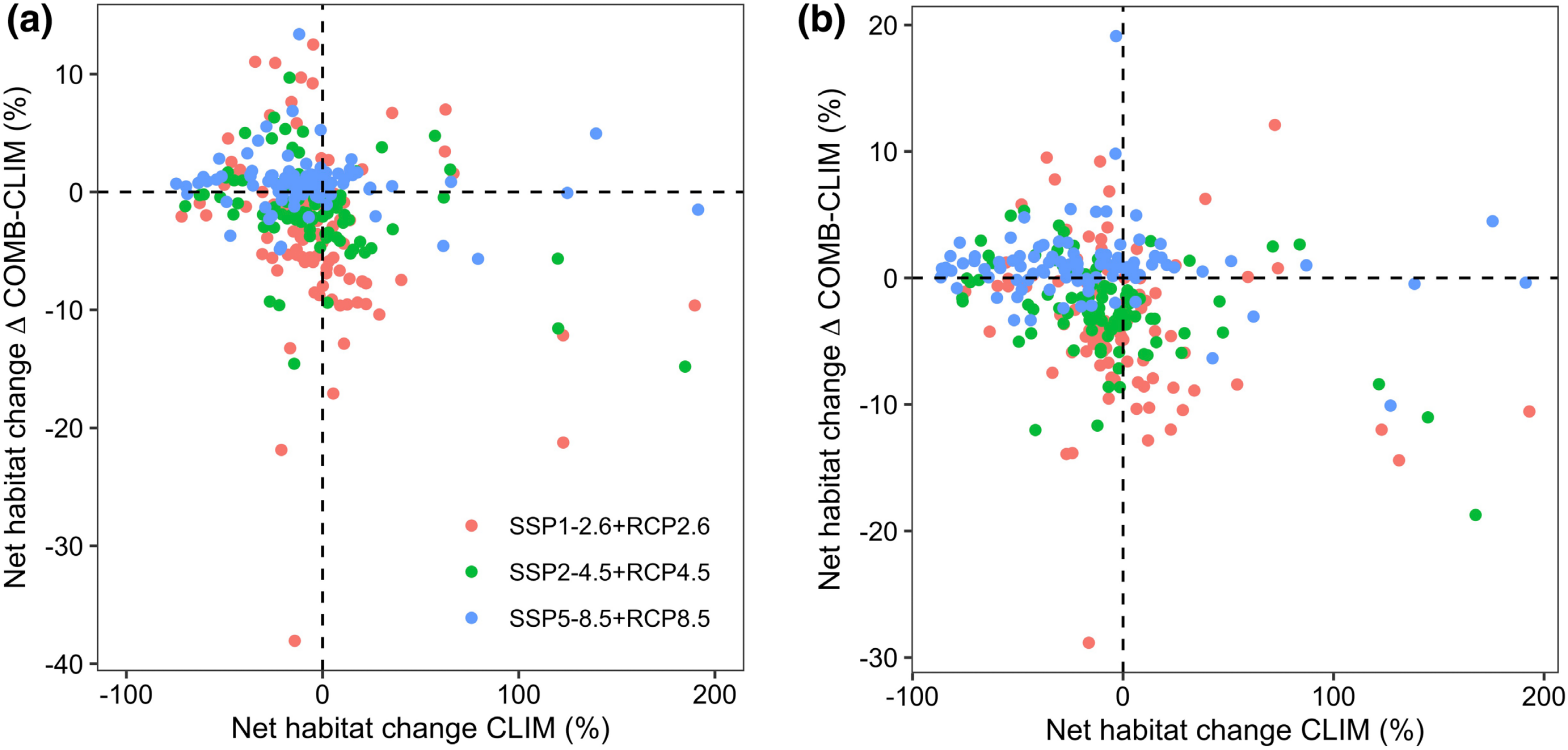
The effect of land use modulation within climate scenarios (i.e., the difference of net habitat changes between COMB [dynamics climate and land use] and CLIM [dynamics climate and constant land use] models) for the 95 Theaceae species under different RCP scenarios by (a) 2050s and (b) 2070s. The points in (a)–(b) represent individual species.

On average, the COMB models predicted more habitat losses than both the CLIM and LU models. Specifically, the mean net habitat changes projected by COMB models is −4.963%, −5.298% and −6.500% for SSP1‐2.6 + RCP2.6, SSP2‐4.5 + RCP 4.5, and SSP5‐8.5 + RCP 8.5 by 2050s, and is −4.831%, −12.080%, and −15.194% by 2070s (Figure 3c). Despite that, incorporating dynamic land use variables into the corresponding climate scenarios also has varying impacts on individual species' response across different scenarios for both time periods, for at least 22 out of the 95 species might gain habitat (Table S3; Figure 3c). Moreover, the dynamic land use variables have an opposite modulating effect on the net habitat change of the 95 Theaceae species (Figure 4). Specifically, 69, 67, and 23 species under SSP1‐2.6 + RCP2.6, SSP2‐4.5 + RCP 4.5 and SSP5‐8.5 + RCP 8.5 by 2050s and 71, 71, and 23 species by 2070s would lose additional habitat compared to the CLIM models, while land use change would offset habitat losses from climate change for the remain species (Figure 5).

## 4 DISCUSSION

In this study, we projected the potential distribution of 95 Chinese Theaceae species under different climate and land use scenarios. By comparing projections with CLIM or LU models, we assessed the isolate effect of climate change and land use change on the future distribution of the 95 Theaceae species. Moreover, an additional projection using COMB models allows us to assess the modulating effect of land use change on distributional shift of an individual species under a certain climate change scenario. According to our knowledge, our study is one of the first studies to assess the future distribution of Theaceae species under the combination of climate change and land use change scenarios, and therefore offers new insights for policy decisions regarding the protection measures for Theaceae species.

Our projections suggested that climate change could have substantial effects on the distributional shift areas of the 95 Theaceae species in China and the climate change impacts will become more intense under higher CO_2_ emission scenarios. Moreover, our projections suggested there is a large variability of the responses of these species to climate change, which is probably related to species‐specific climate requirement and tolerances (Thuiller et al., 2015). These findings are consistent with previous work from Zhang et al. (2020), who assessed future migration patterns and extinction risks of Theaceae species under four emission scenarios (RCP2.6, RCP4.5, RCP6.0 and RCP8.5) at 2070s. However, our results indicate that more species would experience range contractions and less species would experience range expansions under all climate change scenarios, which is largely due to differences in methodology. For example, Zhang et al. (2020) use a coarser spatial resolution (ca. 40 × 40 km) and include constant soil and elevation predictors rather than dynamics land use variables in their SDMs, which could potentially underestimate the magnitude of projected distributional shifts (Escalante et al., 2013; Marshall et al., 2020).

Our projections also suggested that the direction of the isolated effect of land use change on the range shifts of the 95 Theaceae species was complete opposite to the isolated climate impacts. Specifically, the average loss of suitable habitat areas would decrease with increasing greenhouse gas emissions, indicating that better habitat conditions were available for most of the 95 Theaceae species in the future, supporting a general expansion of many Theaceae species to the new suitable habitat in China. Besides, we found that the overall changes in suitable habitat area projected by LU models were smaller than those projected by CLIM models. Consistent with previous studies (e.g., Kerr et al., 2015; Marshall et al., 2018; Soroye et al., 2020), these findings suggest that climate change has rather strong effect while land use change has non‐significant or weak effect for many species distributions at coarse scale. Although in this study the high‐resolution land use data of 10 × 10 km were used, it may still too coarse to sufficiently represent the current and future habitat characters for Theaceae species (Marshall et al., 2020; Préau et al., 2022). As a result, it is different to appreciate the local effects of land use on the future species distributions (Préau et al., 2022). Nonetheless, we also found that there is a large variability of responses to land‐use changes across individual species, which suggests that the land use data we used could to some extent reflect the species‐specific habitat requirements for some of the 95 Theaceae species.

Previous studies have suggested that effects of climate change and land use change on the future species distribution are interactive (i.e., either synergistic or antagonistic), rather than simply additive (Radinger et al., 2016). Consistent with these previous studies, the projections of COMB models suggest that dynamic land use variables have vary modulating effect on the distributional shifts of the 95 Theaceae species, for these Theaceae species show substantial discrepancies in response to the dynamic land use scenarios. Specifically, due to the species‐specific habitat requirement and ecological traits across the 95 Theaceae species, some species would lose additional suitable habitat while other species would gain more suitable habitat when the dynamic land use variables were included in the corresponding climate scenarios. Moreover, these findings also have important conservation implications for Theaceae species. First, land management strategies for the conservation of Theaceae species should take into account those locations that are suitable under current conditions but become unsuitable in future or that are unsuitable under current conditions but become suitable in future (Prestele et al., 2021), and second, for those species may have spatially overlapping habitat, additional cares are required if they response oppositely to land use change when developing land‐based conservation strategies.

Overall, this study provides a comprehensive understanding of the potential isolated and combined impacts of future climate and land use change on the distributional shifts of the 95 Theaceae species. This study also provides useful information for guiding future conservation and management strategies, especially the land‐based management and conservation strategies, for protecting these Theaceae species under future climate and land use change. However, to accurately predict the future species distributions, future research are needed to incorporate diverse ecological processes, such as demographic processes, morphology and dispersal strategies (Meier et al., 2012), and more climate change scenarios (Tang & Zhao, 2022) into the future projections.

## AUTHOR CONTRIBUTIONS

**Junfeng Tang:** Conceptualization (equal); formal analysis (lead); investigation (lead); methodology (equal); project administration (equal); writing – original draft (lead); writing – review and editing (equal). **Xuzhe Zhao:** Conceptualization (equal); funding acquisition (lead); investigation (equal); project administration (lead); resources (lead); supervision (lead); writing – original draft (equal); writing – review and editing (equal).

## CONFLICT OF INTEREST

The authors declare no conflict of interest.

## Supporting information


Tables S1–S3

## Data Availability

The occurrence records of the Theaceae species used in this study are obtain from Zhang et al. (2020), which is available online from the Dryad Digital Repository: https://doi.org/10.5061/dryad.tx95x69td. The climate and land use data used in this study are obtained from Long et al. (2021), which is also available online from the Dryad Digital Repository: https://doi.org/10.5061/dryad.cnp5hqc56.
