# Supplementary material for: Large variability in response to future climate and land‐use changes among Chinese Theaceae species
Source: Ecol Evol. 2022 Nov 15;12(11):e9480. doi: 10.1002/ece3.9480 (PMC9666714; doi:10.1002/ece3.9480)
Supplement: Supplementary file 1 — Tables S1–S3 [file ECE3-12-e9480-s001.docx]

**Type: Research Paper**

**Title: Large variability in response to future climate and land-use changes among Chinese Theaceae species**

**Authors:** Junfeng Tang, Xuzhe Zhao*

**Affiliations**

Key Laboratory of Southwest China Wildlife Resources Conservation (Ministry of Education), Institute of Ecology, China West Normal University, Nanchong, China.

**E-mail addresses of all authors:** Junfeng Tang (junfeng_tang@126.com), Xuzhe Zhao (xuzhe_zhao@126.com)

***To whom correspondence should be addressed:** Xuzhe Zhao, Key Laboratory of Southwest China Wildlife Resources Conservation (Ministry of Education), Institute of Ecology, China West Normal University, 1 Shida Road, Nanchong, 637009, PR China. Email: xuzhe_zhao@126.com

**Supplemental Information**

Tables S1-S3

**Table S1.** Changes in suitable habitats predicted by CLIM models (dynamics climate and constant land use variables) under different future climate change scenarios for the 95 Theaceae species.

| Species | 2050s | | | 2070s | | |
| --- | --- | --- | --- | --- | --- | --- |
|  | SSP2.6 | SSP4.5 | SSP8.5 | SSP2.6 | SSP4.5 | SSP8.5 |
| *Adinandra bockiana* | -14.602 | -14.252 | -18.043 | -17.471 | -22.193 | -35.156 |
| *Adinandra glischroloma* | 13.751 | 10.487 | 1.788 | 14.159 | -0.492 | -26.35 |
| *Adinandra hainanensis* | 20.233 | 17.143 | 27.043 | 24.95 | 31.595 | 61.96 |
| *Adinandra hirta* | 5.46 | 2.559 | -0.967 | 11.863 | -1.509 | -7.96 |
| *Adinandra millettii* | -6.394 | -8.21 | -5.194 | -8.051 | -3.983 | -1.296 |
| *Adinandra nitida* | -28.221 | -30.36 | -32.846 | -24.257 | -28.268 | -24.902 |
| *Anneslea fragrans* | 22.171 | 21.579 | 17.795 | 23.904 | 15.796 | 15.169 |
| *Camellia brevistyla* | -13.21 | -15.922 | -14.664 | -17.711 | -16.725 | -23.492 |
| *Camellia caudata* | 5.189 | 4.375 | 2.478 | 10.736 | 4.411 | 7.9 |
| *Camellia chekiangoleosa* | -9.995 | -13.528 | -17.646 | -10.832 | -15.938 | -46.928 |
| *Camellia cordifolia* | 4.747 | 1.968 | -8.326 | 12.403 | -7.202 | -30.428 |
| *Camellia costei* | -15.489 | -14.309 | -22.376 | -21.86 | -32.131 | -58.898 |
| *Camellia crapnelliana* | 122.62 | 119.925 | 124.77 | 122.823 | 121.545 | 138.448 |
| *Camellia cuspidata* | -13.677 | -13.277 | -15.054 | -11.253 | -13.339 | -22.168 |
| *Camellia drupifera* | 62.698 | 57.294 | 79.252 | 72.109 | 83.9 | 127.06 |
| *Camellia euryoides* | 2.068 | 3.083 | 2.053 | -0.905 | 1.346 | 2.119 |
| *Camellia forrestii* | 66.759 | 61.79 | 61.554 | 59.306 | 45.82 | 42.508 |
| *Camellia fraterna* | -16.427 | -18.831 | -19.043 | -13.747 | -17.592 | -42.269 |
| *Camellia furfuracea* | 22.196 | 19.152 | 23.913 | 29.25 | 29.185 | 37.772 |
| *Camellia grijsii* | 10.933 | 14.201 | 14.725 | 6.435 | 9.965 | 6.233 |
| *Camellia gymnogyna* | -25.667 | -29.89 | -38.352 | -28.112 | -42.65 | -53.364 |
| *Camellia japonica* | -6.549 | -7.247 | -14.162 | -9.484 | -20.904 | -38.373 |
| *Camellia kissii* | 13.926 | 8.954 | 4.176 | 22.762 | 5.747 | 6.037 |
| *Camellia mairei* | -44.046 | -45.053 | -54.121 | -42.958 | -58.45 | -76.054 |
| *Camellia oleifera* | -9.325 | -9.141 | -6.834 | -10.509 | -10.06 | -7.904 |
| *Camellia pitardii* | -13.179 | -12.011 | -23.526 | -16.259 | -31.187 | -48.682 |
| *Camellia polyodonta* | -5.057 | -9.976 | -21.792 | -6.553 | -30.461 | -51.772 |
| *Camellia reticulata* | 35.481 | 30.246 | 15.294 | 39.097 | 13.15 | 5.167 |
| *Camellia rosthorniana* | -24.9 | -24.46 | -35.93 | -29.83 | -45.07 | -70.63 |
| *Camellia salicifolia* | 40.126 | 35.78 | 35.471 | 54.198 | 47.429 | 51.336 |
| *Camellia saluenensis* | 62.355 | 65.095 | 65.49 | 73.549 | 71.133 | 87.065 |
| *Camellia semiserrata* | 122.694 | 120.116 | 139.415 | 131.1 | 144.808 | 175.545 |
| *Camellia sinensis* | -0.072 | 0.135 | 0.881 | -0.716 | -1.825 | -1.846 |
| *Camellia taliensis* | -30.618 | -45.449 | -47.022 | -13.708 | -43.652 | -59.888 |
| *Camellia transarisanensis* | -71.854 | -69.979 | -74.587 | -74.966 | -76.393 | -84.285 |
| *Camellia tsingpienensis* | 8.725 | 2.351 | -3.3 | 7.075 | -11.302 | -21.519 |
| *Camellia yunnanensis* | 20.215 | 15.634 | 11.264 | 22.692 | -2.033 | -10.54 |
| *Cleyera incornuta* | -18.197 | -20.678 | -26.238 | -14.679 | -24.398 | -39.921 |
| *Cleyera japonica* | -9.009 | -11.738 | -14.582 | -7.803 | -14.098 | -21.591 |
| *Cleyera lipingensis* | -41.998 | -40.804 | -48.929 | -55.504 | -63.848 | -78.932 |
| *Cleyera pachyphylla* | -2.578 | -3.759 | -0.977 | 0.365 | 3.115 | 7.744 |
| *Eurya acuminatissima* | 6.053 | 4.303 | 2.128 | 8.114 | 1.428 | -3.783 |
| *Eurya acutisepala* | -8.088 | -11.357 | -20.814 | -10.582 | -26.371 | -50.454 |
| *Eurya alata* | -10.726 | -10.917 | -13.624 | -13.936 | -14.793 | -27.772 |
| *Eurya brevistyla* | 1.894 | 1.46 | -1.913 | -6.965 | -10.608 | -33.606 |
| *Eurya cavinervis* | 9.052 | 7.193 | -1.954 | 8.621 | -9.923 | -16.351 |
| *Eurya chinensis* | 1.602 | 1.456 | 2.406 | 2.018 | 2.253 | 4.631 |
| *Eurya ciliata* | 0.459 | -4.356 | -5.038 | 7.737 | -5.803 | -4.843 |
| *Eurya distichophylla* | 17.832 | 13.479 | 8.014 | 28.396 | 15.008 | 18.225 |
| *Eurya fangii* | -59.31 | -60.59 | -68.968 | -63.361 | -76.211 | -86.55 |
| *Eurya glandulosa* | 8.985 | 7.11 | 3.109 | 2.035 | -1.829 | 5.944 |
| *Eurya groffii* | 10.969 | 9.089 | 2.578 | 15.402 | 2.126 | 2.011 |
| *Eurya handel* | 3.032 | 1.094 | -10.012 | 6.551 | -11.92 | -22.168 |
| *Eurya hebeclados* | -4.825 | -5.267 | -2.612 | -9.585 | -5.462 | -5.708 |
| *Eurya impressinervis* | -39.058 | -43.13 | -52.696 | -46.029 | -61.899 | -77.565 |
| *Eurya japonica* | -11.39 | -12.152 | -18.945 | -13.958 | -20.734 | -42.686 |
| *Eurya kueichowensis* | -24.133 | -24.679 | -35.456 | -32.393 | -47.007 | -63.858 |
| *Eurya loquaiana* | -13.208 | -13.163 | -15.322 | -12.273 | -15.065 | -21.61 |
| *Eurya macartneyi* | -2.733 | -3.798 | 4.505 | -0.819 | 7.965 | 20.772 |
| *Eurya metcalfiana* | -21.009 | -26.858 | -15.294 | -24.068 | -6.869 | -3.595 |
| *Eurya muricata* | -13.296 | -14.264 | -17.029 | -15.984 | -18.942 | -37.829 |
| *Eurya nitida* | -10.12 | -9.973 | -10.041 | -10.768 | -11.474 | -12.834 |
| *Eurya oblonga* | -62.634 | -62.587 | -69.571 | -59.6 | -74.156 | -86.044 |
| *Eurya obtusifolia* | -0.591 | -0.248 | -10.37 | -1.911 | -16.05 | -37.534 |
| *Eurya patentipila* | -10.978 | -15.347 | -25.96 | -10.905 | -28.163 | -50.228 |
| *Eurya pseudocerasifera* | 189.756 | 184.822 | 191.443 | 193.004 | 167.458 | 191.255 |
| *Eurya pyracanthifolia* | -23.097 | -24.715 | -36.843 | -33.561 | -49.504 | -65.794 |
| *Eurya rubiginosa* | -4.066 | -6.458 | -6.009 | -4.015 | -6.216 | -8.072 |
| *Eurya saxicola* | -0.827 | -6.037 | -7.02 | -10.234 | -14.836 | -19.906 |
| *Eurya semiserrulata* | -26.773 | -25.851 | -36.713 | -26.942 | -41.722 | -58.298 |
| *Eurya stenophylla* | -34.343 | -39.503 | -51.461 | -36.254 | -53.344 | -70.393 |
| *Eurya tetragonoclada* | -17.67 | -17.917 | -25.492 | -17.401 | -26.307 | -49.054 |
| *Eurya trichocarpa* | -6.917 | -12.21 | -14.361 | -1.122 | -16.381 | -16.394 |
| *Eurya tsaii* | -19.602 | -22.619 | -29.146 | -22.816 | -35.792 | -48.156 |
| *Eurya weissiae* | -14.167 | -14.451 | -11.912 | -16.269 | -12.174 | -3.328 |
| *Polyspora axillaris* | -12.764 | -18.581 | -20.3 | -7.359 | -17.92 | -17.274 |
| *Polyspora chrysandra* | -4.781 | -16.83 | -21.153 | -7.4 | -28.618 | -43.877 |
| *Polyspora speciosa* | -48.175 | -49.138 | -60.755 | -48.237 | -67.526 | -82 |
| *Pyrenaria hirta* | -10.131 | -11.866 | -16.701 | -8.653 | -14.022 | -29.696 |
| *Pyrenaria microcarpa* | 12.567 | 8.832 | 13.94 | 9.933 | 11.602 | 17.834 |
| *Schima argentea* | -0.639 | 0.226 | -8.204 | 0.645 | -12.667 | -25.411 |
| *Schima brevipedicellata* | -50.089 | -52.126 | -63.312 | -54.56 | -68.508 | -85.625 |
| *Schima crenata* | -15.866 | -18.986 | -27.204 | -10.396 | -23.42 | -28.332 |
| *Schima khasiana* | -16.511 | -22.078 | -27.622 | -26.902 | -41.637 | -52.388 |
| *Schima sinensis* | -46.436 | -48.252 | -58.876 | -54.524 | -72.567 | -81.58 |
| *Schima superba* | -2.935 | -4.463 | -4.765 | 0.128 | -2.066 | -2.437 |
| *Schima wallichii* | -11.158 | -13.49 | -22.4 | -13.009 | -30.329 | -45.133 |
| *Stewartia pteropetiolata* | -1.603 | -6.471 | -13.099 | -6.815 | -23.393 | -34.889 |
| *Stewartia sinensis* | 0.147 | -1.2 | -3.796 | -5.508 | -10.594 | -27.012 |
| *Stewartia villosa* | -30.707 | -29.19 | -28.641 | -30.707 | -19.567 | -12.819 |
| *Ternstroemia gymnanthera* | 10.777 | 11.176 | 10.517 | 15.203 | 13.368 | 13.792 |
| *Ternstroemia kwangtungensis* | 3.329 | -0.355 | -5.924 | -1.554 | -9.706 | -10.612 |
| *Ternstroemia luteoflora* | 5.519 | 2.547 | -3.939 | 9.587 | -1.344 | -1.875 |
| *Ternstroemia microphylla* | -14.798 | -18.381 | -14.399 | -6.967 | -8.825 | -14.997 |
| *Ternstroemia nitida* | 28.942 | 24.861 | 24.328 | 33.846 | 27.767 | 24.316 |

**Table S2.** Changes in suitable habitats predicted by LU models (constant climate and dynamics land use variables) under different future climate change scenarios for the 95 Theaceae species.

| Species | 2050s | | | 2070s | | |
| --- | --- | --- | --- | --- | --- | --- |
|  | SSP2.6 | SSP4.5 | SSP8.5 | SSP2.6 | SSP4.5 | SSP8.5 |
| *Adinandra bockiana* | -1.687 | 0.048 | 0.32 | -1.648 | -0.504 | 0.475 |
| *Adinandra glischroloma* | -5.76 | -1.464 | 0.612 | -5.7 | -2.388 | 0.54 |
| *Adinandra hainanensis* | 3.189 | 1.694 | -1.196 | 2.492 | 1.462 | -1.528 |
| *Adinandra hirta* | -15.861 | -8.538 | 3.962 | -14.552 | -8.892 | 5.236 |
| *Adinandra millettii* | -5.778 | -2.039 | 1.158 | -5.725 | -2.719 | 1.508 |
| *Adinandra nitida* | -4.955 | -2.297 | 3.13 | -3.476 | -2.061 | 4.027 |
| *Anneslea fragrans* | -7.181 | -3.175 | 1.124 | -6.761 | -4.281 | 1.527 |
| *Camellia brevistyla* | -4.761 | -1.692 | 0.456 | -4.924 | -2.592 | 0.521 |
| *Camellia caudata* | 0.224 | 0.161 | -0.913 | -0.009 | -0.421 | -1.27 |
| *Camellia chekiangoleosa* | -5.458 | -1.122 | 2.31 | -5.106 | -2.294 | 2.779 |
| *Camellia cordifolia* | -7.007 | -1.849 | 1.438 | -6.899 | -2.671 | 2.119 |
| *Camellia costei* | 3.349 | 2.729 | 0.198 | 3.323 | 2.669 | 0.215 |
| *Camellia crapnelliana* | -9.129 | -2.711 | -0.358 | -9.254 | -3.832 | -0.405 |
| *Camellia cuspidata* | -5.275 | -2.658 | 0.888 | -5.296 | -3.801 | 1.123 |
| *Camellia drupifera* | 0.076 | -1.55 | -3.137 | -0.265 | -3.59 | -4.308 |
| *Camellia euryoides* | -6.107 | -3.039 | 0.758 | -6.011 | -3.914 | 0.861 |
| *Camellia forrestii* | 1.025 | -1.617 | -3.588 | 0.868 | -3.785 | -5.047 |
| *Camellia fraterna* | -1.207 | -0.085 | 0.773 | -1.186 | -0.731 | 0.816 |
| *Camellia furfuracea* | -6.326 | -2.576 | 0.217 | -6.326 | -3.337 | 0.272 |
| *Camellia grijsii* | -8.089 | -2.41 | 2.673 | -7.756 | -3.348 | 3.641 |
| *Camellia gymnogyna* | -6.906 | -1.571 | 5.216 | -4.283 | -0.459 | 5.676 |
| *Camellia japonica* | -1.225 | -0.519 | -0.266 | -1.178 | -2.422 | -0.492 |
| *Camellia kissii* | -3.099 | -1.184 | -0.054 | -3.261 | -2.615 | -0.506 |
| *Camellia mairei* | -1.195 | -0.155 | 1.023 | -0.933 | -0.581 | 1.432 |
| *Camellia oleifera* | -3.745 | -1.822 | 0.568 | -3.723 | -2.663 | 0.607 |
| *Camellia pitardii* | 4.64 | 2.772 | 0.665 | 4.692 | 3.045 | 0.862 |
| *Camellia polyodonta* | 4.334 | 1.015 | -3.99 | 3.337 | -0.98 | -5.624 |
| *Camellia reticulata* | 7.197 | 3.684 | 0.912 | 7.368 | 5.326 | 1.026 |
| *Camellia rosthorniana* | 1.61 | 0.35 | 1.24 | 1.84 | -0.81 | 1.42 |
| *Camellia salicifolia* | -4.465 | -1.378 | 0.653 | -4.465 | -2.244 | 0.843 |
| *Camellia saluenensis* | 2.067 | 1.951 | -0.325 | 2.113 | 2.81 | -0.348 |
| *Camellia semiserrata* | -8.152 | -3.54 | 2.052 | -7.081 | -3.794 | 2.306 |
| *Camellia sinensis* | -3.764 | -1.605 | 0.567 | -3.756 | -2.579 | 0.711 |
| *Camellia taliensis* | -8.764 | -4.438 | -2.64 | -8.483 | -6.91 | -3.427 |
| *Camellia transarisanensis* | 4.797 | 0.464 | -0.43 | 4.539 | -0.602 | -1.41 |
| *Camellia tsingpienensis* | -8.092 | -2.599 | 0.226 | -8.183 | -3.278 | 0.316 |
| *Camellia yunnanensis* | -2.36 | -1.846 | 0.351 | -1.986 | -2.804 | 0.514 |
| *Cleyera incornuta* | -2.715 | -1.645 | 1.5 | -2.205 | -2.156 | 1.775 |
| *Cleyera japonica* | -5.508 | -2.223 | 0.527 | -5.493 | -3.393 | 0.628 |
| *Cleyera lipingensis* | 3.678 | 1.853 | -0.673 | 3.514 | 1.345 | -0.659 |
| *Cleyera pachyphylla* | -3.211 | -1.042 | 1.654 | -2.76 | -1.342 | 2.212 |
| *Eurya acuminatissima* | -0.274 | 0.057 | -1.04 | -0.492 | -1.059 | -1.674 |
| *Eurya acutisepala* | -0.823 | -0.182 | -0.557 | -0.981 | -1.09 | -1.392 |
| *Eurya alata* | -6.107 | -2.82 | 1.119 | -6.227 | -4.605 | 1.289 |
| *Eurya brevistyla* | -5.858 | -2.75 | 0.9 | -5.764 | -3.951 | 1.227 |
| *Eurya cavinervis* | -1.571 | -0.766 | -0.249 | -1.561 | -1.762 | -0.201 |
| *Eurya chinensis* | -2.184 | -0.881 | 0.527 | -2.233 | -1.81 | 0.541 |
| *Eurya ciliata* | -0.431 | 0.223 | 0.209 | -0.487 | -0.334 | 0.167 |
| *Eurya distichophylla* | -8.027 | -1.608 | 0.719 | -8.053 | -2.772 | 1.072 |
| *Eurya fangii* | 0.405 | 2.479 | 0.632 | 0.745 | 4.456 | 0 |
| *Eurya glandulosa* | -7.956 | -3.086 | -0.366 | -6.973 | -4.184 | -0.754 |
| *Eurya groffii* | -0.066 | -0.008 | 0.107 | -0.14 | -0.649 | -0.082 |
| *Eurya handel* | 4.333 | 2.544 | -0.458 | 4.259 | 3.564 | -0.858 |
| *Eurya hebeclados* | -6.243 | -2.51 | 0.839 | -6.171 | -4.022 | 1.006 |
| *Eurya impressinervis* | -2.203 | -0.942 | 1.783 | -1.739 | -0.522 | 2.652 |
| *Eurya japonica* | -2.518 | 0.29 | 0.853 | -2.874 | -1.201 | 0.994 |
| *Eurya kueichowensis* | 12.009 | 6.786 | 0.508 | 11.895 | 7.218 | 0.648 |
| *Eurya loquaiana* | -4.212 | -1.382 | 0.738 | -4.207 | -2.221 | 0.934 |
| *Eurya macartneyi* | -1.075 | 0.901 | 0.123 | -1.157 | 0.553 | 0.287 |
| *Eurya metcalfiana* | -19.05 | -7.701 | 5.313 | -16.152 | -8.72 | 7.808 |
| *Eurya muricata* | -5.062 | -2.097 | 0.922 | -5.07 | -3.503 | 1.283 |
| *Eurya nitida* | -4.022 | -2.108 | 0.199 | -4.079 | -3.337 | 0.11 |
| *Eurya oblonga* | 2.975 | 3.88 | 1.47 | 3.857 | 5.561 | 1.67 |
| *Eurya obtusifolia* | 3.551 | 1.976 | 0.277 | 3.464 | 1.706 | 0.19 |
| *Eurya patentipila* | 8.356 | 3.477 | -2.094 | 7.81 | 1.912 | -2.749 |
| *Eurya pseudocerasifera* | -9.744 | -6.933 | -4.497 | -9.494 | -8.682 | -5.746 |
| *Eurya pyracanthifolia* | -8.622 | -3.965 | 2.151 | -7.771 | -4.993 | 2.628 |
| *Eurya rubiginosa* | -7.96 | -2.892 | 1.2 | -7.856 | -4.179 | 1.468 |
| *Eurya saxicola* | 2.09 | 1.451 | -2.496 | 1.747 | -1.17 | -3.978 |
| *Eurya semiserrulata* | 9.493 | 6.309 | 1.19 | 9.88 | 9.295 | 1.28 |
| *Eurya stenophylla* | 7.194 | 2.867 | 1.106 | 7.876 | 3.74 | 1.31 |
| *Eurya tetragonoclada* | -5.249 | -1.638 | 1.301 | -5.414 | -2.849 | 1.63 |
| *Eurya trichocarpa* | -2.455 | -0.686 | -0.911 | -2.666 | -1.848 | -1.333 |
| *Eurya tsaii* | -1.617 | -1.282 | -1.518 | -1.4 | -2.938 | -2.386 |
| *Eurya weissiae* | -34.268 | -13.05 | 12.065 | -29.45 | -12.7 | 16.05 |
| *Polyspora axillaris* | -0.206 | -0.499 | -1.278 | 0.059 | -1.234 | -1.748 |
| *Polyspora chrysandra* | 0.72 | 0.917 | -3.209 | 0.917 | -0.982 | -4.977 |
| *Polyspora speciosa* | 8.293 | 4.892 | 2.33 | 9.163 | 6.756 | 3.106 |
| *Pyrenaria hirta* | -3.346 | -0.893 | 0.636 | -3.295 | -1.591 | 0.801 |
| *Pyrenaria microcarpa* | -10.875 | -4.813 | 2.168 | -10.421 | -5.199 | 2.611 |
| *Schima argentea* | -0.161 | 0.187 | 1.038 | -0.026 | -0.019 | 1.348 |
| *Schima brevipedicellata* | 0.745 | 0.387 | 0.646 | 0.993 | 0.378 | 0.954 |
| *Schima crenata* | 4.541 | 3.996 | -1.208 | 3.81 | 2.602 | -1.965 |
| *Schima khasiana* | -18.551 | -12.023 | 2.016 | -17.831 | -15.071 | 2.688 |
| *Schima sinensis* | -0.154 | 0.48 | 1.114 | -0.069 | 0.583 | 1.474 |
| *Schima superba* | -3.931 | -1.79 | 0.316 | -3.85 | -2.706 | 0.464 |
| *Schima wallichii* | -5.403 | -2.813 | 0.389 | -5.098 | -3.988 | 0.361 |
| *Stewartia pteropetiolata* | -8.704 | -4.724 | 2.147 | -7.545 | -5.684 | 1.417 |
| *Stewartia sinensis* | -7.584 | -3.859 | 0.147 | -7.802 | -5.536 | 0.21 |
| *Stewartia villosa* | 0.000 | 1.001 | 2.68 | 2.163 | 1.808 | 2.745 |
| *Ternstroemia gymnanthera* | -3.649 | -1.722 | 0.608 | -3.613 | -2.703 | 0.848 |
| *Ternstroemia kwangtungensis* | -4.051 | -1.652 | 1.2 | -3.794 | -1.775 | 1.261 |
| *Ternstroemia luteoflora* | -4.045 | -1.38 | 0.33 | -3.998 | -1.899 | 0.389 |
| *Ternstroemia microphylla* | 0.265 | 0.796 | -1.228 | -0.63 | -0.829 | -1.559 |
| *Ternstroemia nitida* | -8.186 | -3.536 | 1.066 | -7.98 | -4.517 | 0.957 |

**Table S3.** Changes in suitable habitats predicted by COMB models (dynamics climate and dynamic land use variables) under different future climate change scenarios for the 95 Theaceae species.

| Species | 2050s | | | 2070s | | |
| --- | --- | --- | --- | --- | --- | --- |
|  | SSP2.6 | SSP4.5 | SSP8.5 | SSP2.6 | SSP4.5 | SSP8.5 |
| *Adinandra bockiana* | -15.668 | -13.845 | -17.287 | -17.811 | -21.844 | -33.915 |
| *Adinandra glischroloma* | 6.395 | 7.883 | 3.444 | 6.228 | -3.744 | -23.554 |
| *Adinandra hainanensis* | 22.159 | 18.904 | 24.983 | 25.947 | 32.957 | 58.904 |
| *Adinandra hirta* | -11.627 | -6.828 | 4.292 | -0.979 | -10.142 | -2.7 |
| *Adinandra millettii* | -11.938 | -9.973 | -3.739 | -13.595 | -7.392 | 1.349 |
| *Adinandra nitida* | -32.106 | -32.279 | -28.488 | -30.124 | -31.87 | -19.459 |
| *Anneslea fragrans* | 12.673 | 16.448 | 19.451 | 15.23 | 10.716 | 16.903 |
| *Camellia brevistyla* | -18.08 | -17.874 | -13.275 | -22.354 | -19.447 | -21.562 |
| *Camellia caudata* | 2.246 | 3.275 | 2.505 | 8.938 | 3.087 | 8.133 |
| *Camellia chekiangoleosa* | -14.03 | -14.08 | -14.566 | -17.747 | -19.253 | -42.14 |
| *Camellia cordifolia* | -4.379 | -1.903 | -5.926 | 2.141 | -11.797 | -27.552 |
| *Camellia costei* | -15.127 | -14.025 | -21.842 | -20.362 | -30.719 | -57.632 |
| *Camellia crapnelliana* | 110.469 | 114.27 | 124.692 | 110.827 | 113.148 | 137.981 |
| *Camellia cuspidata* | -18.642 | -15.44 | -13.801 | -16.555 | -16.555 | -20.797 |
| *Camellia drupifera* | 69.69 | 62.056 | 73.583 | 84.203 | 86.546 | 116.969 |
| *Camellia euryoides* | -4.385 | -0.353 | 2.936 | -5.827 | -2.354 | 2.98 |
| *Camellia forrestii* | 68.336 | 61.317 | 56.979 | 59.385 | 43.967 | 36.159 |
| *Camellia fraterna* | -15.113 | -17.729 | -17.846 | -13.334 | -17.602 | -40.595 |
| *Camellia furfuracea* | 14.446 | 14.935 | 24.141 | 23.337 | 24.815 | 38.283 |
| *Camellia grijsii* | -1.926 | 8.976 | 17.499 | -3.913 | 3.944 | 11.185 |
| *Camellia gymnogyna* | -31.254 | -32.854 | -35.077 | -31.772 | -45.139 | -50.178 |
| *Camellia japonica* | -6.855 | -7.081 | -14.162 | -8.465 | -20.511 | -37.775 |
| *Camellia kissii* | 11.537 | 8.233 | 3.971 | 18.166 | 2.701 | 6.037 |
| *Camellia mairei* | -42.54 | -44.071 | -53.065 | -42.769 | -57.64 | -74.908 |
| *Camellia oleifera* | -12.608 | -11.235 | -5.835 | -14.21 | -13.445 | -6.742 |
| *Camellia pitardii* | -7.345 | -8.658 | -22.921 | -12.992 | -28.977 | -47.769 |
| *Camellia polyodonta* | 4.162 | -4.868 | -26.643 | 0.292 | -26.316 | -55.108 |
| *Camellia reticulata* | 42.188 | 34.044 | 16.731 | 45.347 | 16.058 | 6.387 |
| *Camellia rosthorniana* | -25.97 | -25.76 | -34.14 | -32.75 | -47.19 | -69.3 |
| *Camellia salicifolia* | 32.668 | 32.621 | 35.97 | 45.778 | 43.118 | 52.666 |
| *Camellia saluenensis* | 65.792 | 67 | 66.349 | 74.315 | 73.618 | 88.063 |
| *Camellia semiserrata* | 101.452 | 108.551 | 144.372 | 116.685 | 133.787 | 180.029 |
| *Camellia sinensis* | -4.378 | -1.948 | 1.567 | -5.369 | -5.623 | -0.995 |
| *Camellia taliensis* | -30.618 | -47.36 | -50.73 | -16.517 | -48.034 | -61.461 |
| *Camellia transarisanensis* | -73.934 | -71.183 | -73.882 | -76.049 | -77.992 | -83.477 |
| *Camellia tsingpienensis* | 2.057 | 1.379 | -3.639 | -1.175 | -13.675 | -21.745 |
| *Camellia yunnanensis* | 12.643 | 10.75 | 12.409 | 10.703 | -9.184 | -8.67 |
| *Cleyera incornuta* | -20.28 | -22.574 | -24.86 | -15.701 | -25.979 | -37.473 |
| *Cleyera japonica* | -14.95 | -13.968 | -13.405 | -12.474 | -16.978 | -20.898 |
| *Cleyera lipingensis* | -40.118 | -39.83 | -49.767 | -54.282 | -62.092 | -79.742 |
| *Cleyera pachyphylla* | -6.154 | -4.812 | 1.085 | -2.835 | 1.106 | 10.772 |
| *Eurya acuminatissima* | 5.901 | 3.972 | 1.059 | 8.086 | 0.019 | -5.75 |
| *Eurya acutisepala* | -9.251 | -10.643 | -21.504 | -11.2 | -26.19 | -50.611 |
| *Eurya alata* | -16.217 | -13.454 | -12.682 | -19.157 | -18.93 | -27.148 |
| *Eurya brevistyla* | -5.002 | -2.089 | -0.912 | -13.666 | -16.221 | -31.926 |
| *Eurya cavinervis* | 6.695 | 5.862 | -2.404 | 6.245 | -11.485 | -16.849 |
| *Eurya chinensis* | -2.205 | -0.305 | 3.134 | -1.338 | -0.652 | 5.477 |
| *Eurya ciliata* | -0.793 | -4.954 | -4.884 | 6.401 | -6.652 | -4.704 |
| *Eurya distichophylla* | 8.459 | 11.113 | 9.583 | 17.95 | 11.779 | 19.833 |
| *Eurya fangii* | -61.287 | -60.801 | -69.13 | -67.607 | -78.059 | -86.501 |
| *Eurya glandulosa* | -0.64 | 3.269 | 3.498 | -4.572 | -7.682 | 4.047 |
| *Eurya groffii* | 10.09 | 8.801 | 2.742 | 14.204 | 1.141 | 2.323 |
| *Eurya handel* | 5.738 | 2.484 | -10.204 | 8.844 | -10.426 | -22.079 |
| *Eurya hebeclados* | -10.765 | -7.603 | -1.432 | -15.395 | -9.549 | -4.717 |
| *Eurya impressinervis* | -40.304 | -44.087 | -49.87 | -46.71 | -60.725 | -74.768 |
| *Eurya japonica* | -12.293 | -10.636 | -18 | -15.159 | -20.593 | -42.346 |
| *Eurya kueichowensis* | -13.191 | -18.363 | -34.909 | -24.603 | -41.695 | -63.807 |
| *Eurya loquaiana* | -18.528 | -15.311 | -14.147 | -16.799 | -17.275 | -20.491 |
| *Eurya macartneyi* | -5.17 | -4.453 | 5.283 | -2.948 | 6.317 | 21.806 |
| *Eurya metcalfiana* | -42.876 | -36.142 | -8.425 | -37.913 | -15.482 | 6.225 |
| *Eurya muricata* | -18.196 | -16.199 | -15.262 | -20.109 | -21.008 | -35.218 |
| *Eurya nitida* | -13.875 | -11.683 | -9.534 | -14.738 | -14.361 | -12.787 |
| *Eurya oblonga* | -63.574 | -62.857 | -69.101 | -60.223 | -74.203 | -85.303 |
| *Eurya obtusifolia* | 2.275 | 1.145 | -9.939 | -1.028 | -16.422 | -37.111 |
| *Eurya patentipila* | -1.274 | -11.597 | -27.999 | -1.693 | -24.522 | -51.739 |
| *Eurya pseudocerasifera* | 180.137 | 170.019 | 189.944 | 182.448 | 148.72 | 190.881 |
| *Eurya pyracanthifolia* | -29.755 | -27.726 | -35.403 | -41.06 | -54.545 | -65.093 |
| *Eurya rubiginosa* | -12.587 | -9.643 | -4.515 | -12.069 | -10.127 | -6.242 |
| *Eurya saxicola* | 0.998 | -4.571 | -9.158 | -7.941 | -15.382 | -22.153 |
| *Eurya semiserrulata* | -20.256 | -21.307 | -35.373 | -23.123 | -39.917 | -57.514 |
| *Eurya stenophylla* | -23.301 | -34.494 | -50.137 | -26.74 | -48.417 | -68.687 |
| *Eurya tetragonoclada* | -22.979 | -20.16 | -24.086 | -23.211 | -29.081 | -47.334 |
| *Eurya trichocarpa* | -8.646 | -12.83 | -14.955 | -3.524 | -17.912 | -17.437 |
| *Eurya tsaii* | -20.726 | -21.968 | -30.448 | -25.34 | -37.113 | -49.083 |
| *Eurya weissiae* | -52.222 | -29.012 | 1.467 | -45.106 | -23.845 | 15.787 |
| *Polyspora axillaris* | -12.603 | -18.258 | -20.505 | -6.713 | -17.626 | -17.347 |
| *Polyspora chrysandra* | 7.728 | -7.138 | -25.802 | -3.405 | -27.636 | -47.217 |
| *Polyspora speciosa* | -43.64 | -47.911 | -59.481 | -42.429 | -64.575 | -80.276 |
| *Pyrenaria hirta* | -13.796 | -14.176 | -15.397 | -12.061 | -16.311 | -28.423 |
| *Pyrenaria microcarpa* | 3.031 | 4.688 | 15.859 | 1.351 | 5.494 | 20.524 |
| *Schima argentea* | 0.058 | 0.948 | -6.862 | 0.645 | -11.951 | -24.341 |
| *Schima brevipedicellata* | -49.473 | -52.563 | -62.547 | -55.216 | -68.677 | -85.267 |
| *Schima crenata* | -8.232 | -13.648 | -29.501 | -7.342 | -20.884 | -30.722 |
| *Schima khasiana* | -29.758 | -31.677 | -26.71 | -40.821 | -53.66 | -51.02 |
| *Schima sinensis* | -43.883 | -46.59 | -57.968 | -54.592 | -72.892 | -81.014 |
| *Schima superba* | -8.366 | -6.596 | -3.789 | -4.759 | -5.028 | -0.855 |
| *Schima wallichii* | -14.998 | -15.849 | -22.372 | -17.052 | -32.726 | -45.3 |
| *Stewartia pteropetiolata* | -10.351 | -10.222 | -12.326 | -16.349 | -29.12 | -35.82 |
| *Stewartia sinensis* | -7.816 | -5.893 | -3.431 | -13.387 | -16.474 | -26.97 |
| *Stewartia villosa* | -35.97 | -30.772 | -23.087 | -30.965 | -21.117 | -7.588 |
| *Ternstroemia gymnanthera* | 6.403 | 9.239 | 11.232 | 10.997 | 10.144 | 14.865 |
| *Ternstroemia kwangtungensis* | -2.521 | -2.411 | -5.08 | -5.912 | -10.575 | -9.535 |
| *Ternstroemia luteoflora* | -0.047 | 0.743 | -3.16 | 3.078 | -3.974 | -0.767 |
| *Ternstroemia microphylla* | -18.149 | -19.21 | -15.627 | -7.764 | -9.456 | -17.186 |
| *Ternstroemia nitida* | 18.552 | 20.09 | 24.691 | 24.946 | 21.845 | 25.151 |
